# Efficacy of MRI in primary care for patients with knee complaints due to trauma: protocol of a randomised controlled non-inferiority trial *(TACKLE trial)*

**DOI:** 10.1186/1471-2474-15-63

**Published:** 2014-03-03

**Authors:** Nynke M Swart, Kim van Oudenaarde, Paul R Algra, Partick JE Bindels, Wilbert B van den Hout, Bart W Koes, Rob GHH Nelissen, Jan AN Verhaar, Hans JL Bloem, Sita MA Bierma-Zeinstra, Monique Reijnierse, Pim AJ Luijsterburg

**Affiliations:** 1 Department of General Practice, Erasmus MC, University Medical Center Rotterdam, PO Box 2040, 3000, CA, Rotterdam, The Netherlands; 2 Department of Radiology, Leiden University Medical Centre, PO Box 9600, 2300, RC, Leiden, The Netherlands; 3 Department of Radiology, Medical Centre Alkmaar, Alkmaar, Wilhelminalaan 12, 1815, JD, Alkmaar, The Netherlands; 4 Department of Medical Decisions, Leiden University Medical Centre, PO Box 9600, 2300, RC, Leiden, The Netherlands; 5 Department of Orthopaedics, Leiden University Medical Centre, PO Box 9600, 2300, RC, Leiden, The Netherlands; 6 Department of Orthopaedics, Erasmus MC, University Medical Center Rotterdam, PO Box 2040, 3000, CA, Rotterdam, The Netherlands

**Keywords:** Traumatic knee complaint, General practice, Magnetic resonance imaging, Randomised controlled non-inferiority trial, Cost-utility, Cost-effectiveness

## Abstract

**Background:**

Patients with traumatic knee complaints regularly consult their general practitioner (GP). MRI might be a valuable diagnostic tool to assist GPs in making appropriate treatment decisions and reducing costs. Therefore, this study will assess the cost-effectiveness of referral to MRI by GPs compared with usual care, in patients with persistent traumatic knee complaints.

**Design and methods:**

This is a multi-centre, open-labelled randomised controlled non-inferiority trial in combination with a concurrent observational cohort study. Eligible patients (aged 18–45 years) have knee complaints due to trauma (or sudden onset) occurring in the preceding 6 months and consulting their GP. Participants are randomised to: 1) an MRI group, i.e. GP referral to MRI, or 2) a usual care group, i.e. no MRI. Primary outcomes are knee-related daily function, medical costs (healthcare use and productivity loss), and quality of life. Secondary outcomes are disability due to knee complaints, severity of knee pain, and patients’ perceived recovery and satisfaction. Outcomes are measured at baseline and at 1.5, 3, 6, 9 and 12 months follow-up. Also collected are data on patient demographics, GPs’ initial working diagnosis, GPs’ preferred management at baseline, and MRI findings.

**Discussion:**

In the Netherlands, the additional diagnostic value and cost-effectiveness of direct access to knee MRI for patients presenting with traumatic knee complaints in general practice is unknown. Although GPs increasingly refer patients to MRI, the Dutch clinical guideline ‘Traumatic knee complaints’ for GPs does not recommend referral to MRI, mainly because the cost-effectiveness is still unknown.

**Trial registration:**

Dutch Trial Registration: NTR3689.

## Background

General practitioners (GPs) are often consulted by patients with traumatic knee complaints. For musculoskeletal disorders, knee complaints are the second most frequent reason (after low back pain) for consulting the GP [1]. Traumatic knee complaints are knee complaints due to a trauma of the knee or are at least of a sudden onset, and therefore likely to be traumatic. Traumatic knee complaints can be caused by e.g. bone bruise, fracture, and/or soft tissue injuries such as lesions of menisci, cruciate ligaments, collateral ligaments and muscles [2-4]. In Dutch general practice, the incidence and prevalence of knee complaints are estimated at 20 and 30 per 1000 persons/year, respectively, whereas the incidence and prevalence of traumatic knee complaints are estimated at 5.3 and 6.8 per 1000 persons/year, respectively [1].

For the GP, diagnosing knee injuries other than fracture or locked knee can be difficult [5-8]. Magnetic resonance imaging (MRI) of the knee can help in establishing the correct diagnosis or in excluding other diagnoses; this additional knowledge can be used to decide on subsequent treatment and/or referral of patients with traumatic knee complaints. MRI is a powerful diagnostic tool for detecting lesions of ligaments, tendons, bone, cartilage and menisci [4,9,10]. MRI showed a sensitivity of 86%, 91%, 76%, a specificity of 95%, 81%, 93% and an accuracy of 93%, 86%, 89% for anterior cruciate ligament, medial and lateral meniscus lesions, respectively [9].

Recommendations for the diagnosis and management of patients with traumatic knee complaints presenting in primary care in the Netherlands are described in the clinical guideline ‘Traumatic knee complaints’ issued by the Dutch College of General Practitioners in 2010 [2]. At the GPs’ initial consultation an urgent referral to a medical specialist is required when there are signs of a fracture, acute locked knee, or severe complaints after patella dislocation [2]. Otherwise, patients are managed conservatively; this generally comprises information and advice about the knee complaints, medication for pain reduction and, if indicated, referral to physical therapy. When complaints have not decreased at follow-up the GP can refer the patient to an orthopaedic surgeon who may request an MRI or perform an arthroscopy or surgery [11]. In the Netherlands, at 1-year follow-up, 57% of patients with traumatic knee complaints had consulted their GP more than once, about one third was referred to physical therapy, and 21% were referred to an orthopaedic surgeon [12].

Direct referral to MRI might be a valuable tool for GPs in making appropriate and informed decisions [13]. Negative MRI findings may enable the GP to reassure patients, treat them conservatively, and avoid unnecessary orthopaedic referrals. Positive MRI findings could confirm the GP’s diagnosis and the decision to either advise conservative treatment or refer to an orthopaedic surgeon in an earlier stage [14].

The DAMASK trial showed that an MRI referral by the GP prior to a provisional orthopaedic appointment yielded significant benefits in patients’ knee-related quality of life when compared with direct referral to an orthopaedic surgeon [15]. Another study showed that early MRI of the knee in patients in secondary care with suspected internal derangement facilitates faster diagnosis at a comparable cost level compared with physical therapy; at 3-months follow-up patients randomised for an early MRI reported significantly less pain, less activity limitations and better patient satisfaction [16].

### Aim

Whether MRI of the knee should enter the diagnostic pathway in primary care, through direct access by GP’s, depends on whether it improves patient outcomes, reduces costs and affects subsequent diagnosis and management. The objectives of this study over a period of 12 months follow-up are:

1. To assess the cost-effectiveness of MRI referral by the general practitioner compared to usual care in patients with persistent traumatic knee complaints.

2. To assess if MRI referral by the general practitioner is noninferior compared to usual care in patients with persistent traumatic knee complaints regarding self-reported knee related daily function.

## Methods

This study has been approved by the Medical Ethics Committee of the Erasmus Medical Centre (Dutch Trial Registration: NTR3689) [17].

### Design

**Figure 1:**
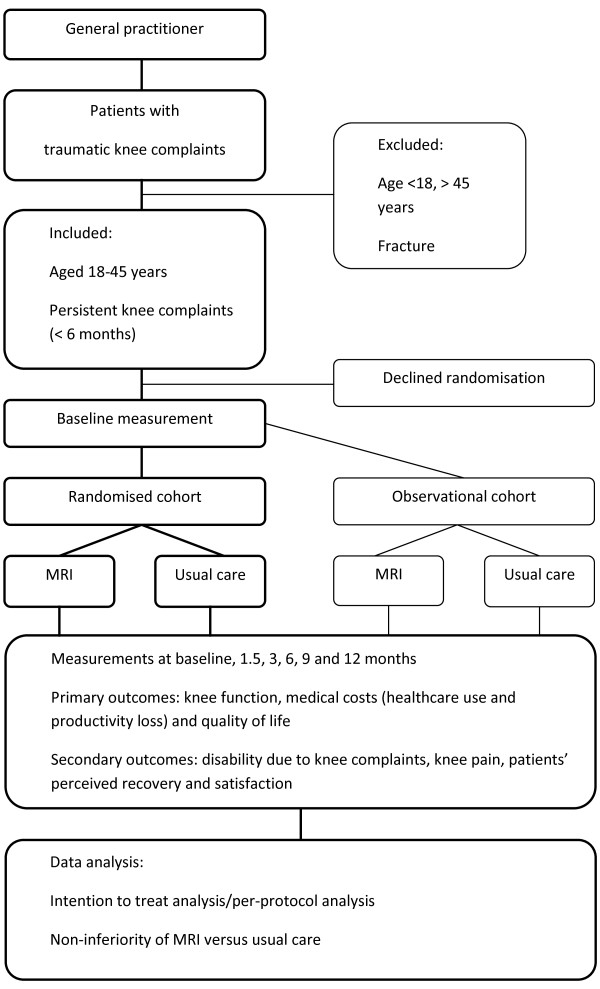
Flow chart of the study.

The study will be a multi-centre, parallel group, open-labelled, non-inferiority randomised controlled trial (RCT) with a 1-year follow-up. Figure 1 presents a flow chart of the study.

To assess the generalisability of the findings, patients who are eligible but decline randomisation are invited to participate in the concurrent observational cohort study; in this latter study the inclusion criteria and measurements are identical to those for the randomised patients. Inclusion of these latter patients in an observational cohort will provide insight into the potential selection of patients entering the randomised cohort. Furthermore, it allows to assess the course (e.g. medical consumption and outcomes) of these non-randomised patients presenting with knee complaints after a trauma within the participating general practices, including the frequency of MRI referral and referral to an orthopaedic surgeon.

### Study population

GPs located in the south west area of the Netherlands will recruit eligible patients. The GP informs the patient and sends contact data to the researchers. The researcher contacts the patient by telephone and checks the inclusion/exclusion criteria.

Patients are eligible for inclusion if they (re)consulted their GP with knee complaints (knee pain and/or disability) due to trauma or sudden onset in the preceding 6 months and are aged 18–45 years. Patients are excluded if there is an indication for direct referral to an orthopaedic surgeon (e.g. fracture, acute locked knee, or severe complaints after patella dislocation).

Patients are also excluded when: 1) the knee complaints are already managed in secondary care, 2) the patient is known with osteoarthritis in the affected knee (diagnosis confirmed by a medical specialist), 3) there is other non-traumatic arthropathy (e.g. infection, Reiter’s syndrome, gout, inflammatory bowel disease, or neuropathic pain) or isolated patellofemoral joint pain, 4) there is a previous MRI of the knee within the same episode of knee complaints, 5) there is a previous surgical intervention of the affected knee, or 6) there are contra-indications for MRI (e.g. claustrophobia, metal implants or pregnancy).

### Randomisation and interventions

When patients are eligible for inclusion and have completed the informed consent procedure, the baseline measurement will take place. Hereafter, patients are randomly allocated to the MRI or the usual care group. An independent person produces a randomisation list by computer, using random blocks of 4 and 6. Allocation by one of the researchers (KvO or NS) will be concealed and cannot be influenced or predicted because the randomisation list is not accessible to members of the research team.

#### MRI group

Patients will be referred for an MRI scan of the affected knee at one of the participating MRI centres (in Rotterdam, Amsterdam, Alkmaar, Goes, or Leiden) within 2 weeks after referral. MRI is performed on a 1.5 T system using the ‘acute knee scanning protocol’; this is available in all participating centres and is adjusted for the specific magnetic resonance device. All protocols include imaging in the coronal, sagittal and transversal plans, and all include a T1 and a PD-weighted sequential, with or without fat suppression. All participating musculoskeletal radiologists (n = 12) have adequate experience working with these predefined protocols.

In the Netherlands, there is no standardised way for a radiologist to score and report MRI findings for patients with traumatic knee problems. For this reason, a standardised and a digitalised report was developed for the TACKLE Trial. This report was composed as an online questionnaire, using an open source survey application called the Lime Survey [18]. All radiologists are trained in this standardised scoring of MRI features.

The following items are scored in the MRI report: the quantity of synovial fluid and soft tissues, menisci, anterior and posterior cruciate ligaments, medial and lateral collateral tendons and the bone and cartilage. The report will produce a treatment/referral advice for the GP based on the latest consensus in the literature, expert opinion and daily practice [11,19]. Table 1 presents an overview of the most significant findings and the treatment/referral advice for GPs.

**Table 1:** Types of findings on MRI and related advice

**Positive findings (advice for referral to orthopaedic surgeon)**	**Equivocal findings (advice based on radiologist’s judgement)**	**Negative findings (advice for treatment in primary care)**
Pigmented villonodular synovitis	Synovitis, bursitis, hoffitis, any other cyst	Effusion, Baker’s cyst, ganglion, plica, subcutaneous oedema
	Lesions of the m. quadriceps tendon, the patellar tendon or the patellar retinacula	
Osteochondrosis dissecans fracture	Lesions of the trochlea or patellar alignment anomalies	Bone bruise or bone marrow oedema
Meniscal tears*		Parameniscal cyst, meniscal extrusion, discoid meniscus, isolated lesions of meniscal ligaments or meniscal capsular lesions
Partial or complete anterior or posterior cruciate ligament tears		Mucoid degeneration of the cruciate ligaments
Grade III injury (complete rupture) of the medial collateral ligament or the posterolateral corner		Grade I and II injury of the medial collateral ligament or the posterolateral corner
Grade IV chondromalacia		Grade I to III chondromalacia

*A meniscal tear is defined as an abnormal shape of the meniscus OR as a high signal intensity unequivocally contacting the surface of the meniscus. The latter must be seen on at least 2 adjacent slices in one plane.

The radiologist will report the details on possible pathology to the GP, together with a treatment/referral advice (based on Table 1). In case of positive MRI findings, the advice of the radiologist will be to refer to an orthopaedic surgeon. The orthopaedic surgeon will decide whether arthroscopy or surgery is required, based on clinical findings and on the Dutch orthopaedic guidelines [11,19]. In case of negative MRI findings the advice of the radiologist will be to continue treatment in primary care according to the Dutch clinical guideline ‘Traumatic knee complaints’ (see Usual care group). In case of equivocal findings, based on severity of the injury, the radiologist will decide whether the advice will be to continue treatment in primary care or to refer to an orthopaedic surgeon. Finally, the GP will decide whether or not to refer the patient, based on the radiologist’s report and the patient’s current complaints.

The inter-rater reliability of the radiologist’s advice was determined for eight participating radiologists using 10 MRIs of patients with traumatic knee complaints. The intra-class correlation coefficient was 0.65, reflecting reasonable agreement.

#### Usual care group

These patients are treated according to the Dutch clinical guideline ‘Traumatic knee complaints’, i.e. without MRI [2]. When there are signs of contusion, distortion, medial or lateral collateral ligament lesion, patients are advised to continue their daily activities and load the knee as much as possible. When there are indications of meniscal lesions and/or cruciate ligament lesions, patients are advised to take rest for a few days and to use elbow crutches if necessary. When pain and effusion decreases patients are advised to flex and extend the knee without load bearing, to do isometric muscle training of the quadriceps muscle, and gradually increase their daily activities. For additional support regarding exercises the GP can refer the patient to a physical therapist. Follow-up consultations are planned with an interval of (at most) 2 weeks.

### Outcomes

Patients will fill in questionnaires at baseline and at 1.5, 3, 6, 9 and 12-months follow-up (Table 2). The questionnaires are sent by e-mail which contains a secured hyperlink to the questionnaire. For this purpose the survey application the Lime Survey is used [18].

**Table 2:** Measurement of primary and secondary outcomes

	**Baseline**	**1.5 months**	**3 months**	**6 months**	**9 months**	**12 months**
Primary						
Lysholm	X	X	X	X	X	X
iMCQ/iPCQ	X	--	X	X	X	X
EQ-5D-3 L	X	X	X	X	X	X
Secondary						
KOOS	X	X	X	X	X	X
NRS	X	X	X	X	X	X
GPE	X	X	X	X	X	X
Satisfaction	X	X	X	X	X	X

Lysholm = Lysholm Scale. iMCQ = Medical Consumption Questionnaire from the Institute for Medical Technology Assessment. iPCQ = Productivity Cost Questionnaire from the Institute for Medical Technology Assessment. EQ-5D-3 L = EuroQol 5-Dimensions. KOOS = Knee Injury and Osteoarthritis Outcome Score. NRS = numeric rating scale. GPE = Global Perceived Effect.

#### Primary outcomes

1) Patients’ knee-related daily function is measured with the Lysholm Scale [20]. This scale is well documented according to validity, reliability and responsiveness in patients with traumatic knee injuries [21,22]. The Lysholm Scale summarizes activity limitations and symptoms related to activity. The score consists of 8 items rated on a 100-point scale, with instability and pain being allocated 25 points each [20]. A higher score indicates better knee function.

2) Medical costs are measured for the health care use and productivity loss. Healthcare use is measured with the Medical Consumption Questionnaire from the Institute for Medical Technology Assessment (iMCQ), adjusted to fit our population [23]. The iMCQ includes questions related to frequently occurring contacts with healthcare providers. Healthcare costs are calculated by multiplying healthcare use by Dutch standard prices [24].

Productivity loss is measured with the Productivity Cost Questionnaire from the Institute for Medical Technology Assessment (iPCQ) [25]. The iPCQ consist of 12 items in three modules: lost productivity at paid work due to absenteeism, lost productivity at paid work due to presenteeism, and lost productivity at unpaid work. Productivity costs are calculated by multiplying productivity losses by standard Dutch age and sex-specific prices per hour [24].

3) Patients’ quality of life is measured with the EuroQol 5-Dimensions (EQ-5D-3 L). The EQ-5D-3 L consists of 6 items. Items 1–5 measure the health state on five dimensions (mobility, self-care, usual activities, pain/discomfort, and anxiety/depression). Each dimension has 3 levels: level 1 indicates no problems, level 2 indicates some problems, and level 3 indicates extreme problems. Item 6 measures the self-rated health on a vertical visual analogue scale (VAS) where the endpoints are labelled best imaginable health state (100) and worst imaginable health state (0). [26] There is evidence of construct validity and reliability for patients with knee injuries [27].

#### Secondary outcomes

1) Disability due to knee complaints is assessed with the Knee Injury and Osteoarthritis Outcome Score (KOOS) [28]. This questionnaire consist of 42 questions for five dimensions (pain, symptoms, function in daily living, function in sport and recreation, and knee-related quality of life). The answer options are standardised and rated on a scale from 0–4. The total score is calculated for each subscale on a scale from 0–100, a higher score indicating more symptoms. The KOOS has good validity, reliability, responsiveness, internal consistency and no floor or ceiling effect [28].

2) Severity of knee pain is assessed with the numeric rating scale (NRS). The NRS is an 11-point Likert scale, where 0 indicates no pain and 10 indicates unbearable pain. The NRS is a valid, reliable and appropriate rating scale for capturing severity of pain in clinical practice [29].

3) Patients’ perceived recovery is assessed with the Global Perceived Effect (GPE). The GPE is a 7-point Likert scale ranging from completely recovered to worse than ever [30]. The reliability of the GPE is excellent [31].

4) Patients’ satisfaction with the treatment is measured on a 7-point Likert scale ranging from absolutely satisfied to absolutely dissatisfied.

At baseline the following demographic data are collected: age, gender, height, weight, education level, co-morbidity, duration of complaints and previous knee complaints. Also collected are data on GPs’ initial working diagnosis, GPs’ preferred management at baseline, and MRI findings.

### Sample size calculation

The sample size is based on the Lysholm Scale. In our pilot study, at 1-year follow-up, the effect (Lysholm Scale) of usual care in general practice was estimated at a mean difference of −23 with a standard deviation of 17 (95% confidence interval; CI −27.8; −18.2) [12]. To obtain 80% statistical power with a 2-sided alpha of 0.05, 225 patients per treatment group are required to establish the non-inferiority of MRI referral by the GP compared with usual care within 4.8 points on the Lysholm Scale. Hence, using a 2-sided alpha of 0.05 and 225 patients per group, the trial has a 91% power to detect superiority of MRI referral over usual care assuming a clinically relevant difference of 15% in knee function. Based on previous studies we expect a loss to follow-up of 15%; therefore, the planned trial will require 520 patients with traumatic knee complaints [12,14].

### Statistical analysis

Success of the randomisation and distribution of outcome measures will be checked before the actual analyses are performed. The baseline characteristics of the non-randomised patients in the cohort are analysed and compared with those of the randomised patients to gain insight into potential selection bias.

The economic evaluation is a cost-utility analysis from the societal perspective (costs per quality adjusted life-year; QALY), based on patients’ reports. A 1-year time horizon will be used, without discounting. Costs related to outcome are analysed using net-benefit acceptability curves, multiple imputation and bootstrapping, including only the uncertainty due to trial sampling error. Cost price analyses are performed for MRI and orthopaedic consultations. Other costs are valued using standard prices (including time involved and travel costs) [24]. QALYs are estimated as the area under the observed 1-year utility curves. Utilities are estimated using the EQ-5D-3 L (primary analysis, Dutch tariff) and the patients’ health VAS, transformed to a utility scale using the power transformation U = 1-(1-VAS/100)^1.61^.

We will evaluate whether MRI referral by GPs is non-inferior compared with usual care in accordance with the clinical guideline, beyond a specified non-inferiority margin (delta) with a defined confidence interval. Non-inferiority of MRI over usual care will be accepted if the upper bound of the 95% CI around the estimated difference in primary outcome (Lysholm Scale) lies below delta. A delta of 4.8 is adopted; this is based on the expected effect in the usual care group as found in our pilot study (see Sample size calculation), and on judgement about the difference between treatments that would be clinically meaningful.

The outcome of both groups are analysed on the basis of the ‘intention to treat’ principle. Linear mixed models with repeated measurements are used to calculate group differences over time. We will adjust for baseline variables that have a clinically meaningful difference between the two groups. In non-inferiority trials, because an intention to treat analysis can increase the type I error (i.e. the risk of falsely claiming non-inferiority), we will also perform a per-protocol analysis [32].

Additionally, we will perform exploratory analysis to identify clinical indicators for better (cost) effectiveness over a 1-year period using univariable and multivariate logistic regression analysis. Different usual thresholds (i.e. 16, 20 and 40 thousand euros per QALY) for the maximum willingness to pay for an extra QALY will be explored.

## Discussion

Although GPs in the Netherlands increasingly refer patients with knee complaints to MRI, there is lack of evidence regarding whether or not this is cost-effective care. We have reported the design of a non-inferior RCT to investigate the cost-effectiveness of MRI on referral of the GP compared with usual care, in patients with traumatic knee complaints.

## Competing interest

The authors declare that they have no competing interests.

## Authors’ contribution

All authors made substantive intellectual contributions to this research protocol. MR, PL, PA, SBZ, PB, JB, WvdnH, BK, RN and JV conceptualized the primary research questions and constructed the study design. KvO and NS contributed to the implementation of the study design into the current state of the trial. MR, PL, KvO and NS co-ordinate the trial. KvO and NS are responsible for writing this article which is based on the funding application written by PL, MR, SBZ, JB, WvdnH, PA, BK, PB, RN and JV and the medical ethical approval application, written by NS, KvO, PL and MR. All authors have participated sufficiently in this work to take public responsibility for their portions of the content. All authors read and approved the final manuscript.

## Pre-publication history

The pre-publication history for this paper can be accessed here:

http://www.biomedcentral.com/1471-2474/15/63/prepub
